# Influence of Synthesis Conditions on Catalytic Performance of Ni/CeO_2_ in Aqueous-Phase Hydrogenolysis of Glycerol without External Hydrogen Input

**DOI:** 10.3390/molecules29163797

**Published:** 2024-08-10

**Authors:** Clara Jarauta-Córdoba, Lucía García, Joaquín Ruiz, Miriam Oliva, Jesús Arauzo

**Affiliations:** 1 CIRCE-Energy Resources and Consumption Technology Center, Parque Empresarial Dinamiza, Avda. Ranillas 3D, 1st Floor, 50018 Zaragoza, Spain; cajarauta@fcirce.es; 2 Thermochemical Processes Group (GPT), Aragon Institute of Engineering Research (I3A), Universidad de Zaragoza, Mariano Esquillor S/N, 50018 Zaragoza, Spain; jruizp@unizar.es (J.R.); miroliva@unizar.es (M.O.); jarauzo@unizar.es (J.A.)

**Keywords:** Ni/CeO_2_ catalyst, glycerol, hydrogenolysis, 1,2-propanediol, acetol

## Abstract

The aqueous-phase hydrogenolysis of glycerol was studied in Ni/CeO_2_ catalytic systems prepared by incipient wetness impregnation. The operating conditions were 34 bar, 227 ºC, 5 wt.% of glycerol, and a W/m_glycerol_ = 20 g catalyst min/g glycerol without a hydrogen supply. The effect of the catalyst preparation conditions on the catalytic activity and physicochemical properties of the catalysts was assessed, particularly the calcination temperature of the support, the calcination temperature of the catalyst, and the Ni content. The physicochemical properties of the catalysts were determined by N_2_ adsorption, H_2_-TPR, NH_3_-TPD, and XRD, among other techniques. A relevant increase in acidity was observed when increasing the nickel content up to 20 wt.%. The increase in the calcination temperatures of the supports and catalysts showed a detrimental effect on the specific surface area and acid properties of the catalysts, which were crucial to the selectivity of the reaction. These catalysts notably enhanced the yield of liquid products, achieving global glycerol conversion values ranging from 17.1 to 29.0% and carbon yield to liquids ranging from 12.6 to 24.0%. Acetol and 1,2-propanediol were the most abundant products obtained in the liquid stream.

## 1. Introduction

At present, transportation fuels are predominantly derived from fossil oil, a situation deemed unsustainable due to the associated emissions of greenhouse gases contributing to climate change. The finite nature of oil reserves exacerbates this concern [1]. The absence of robust models for forecasting oil depletion [2], coupled with emerging policy frameworks advocating for the decarbonization of this sector, has led to the integration of biofuels as among the most promising options to attain these objectives. Particularly, biodiesel arose as a great alternative in the market for diesel engines. Its production from organic feedstocks such as vegetable oils, animal fats, or algae mitigates greenhouse gas emissions, counteracting global warming and diversifying energy sources, enhancing energy security, and promoting rural development through new opportunities for local farmers and communities engaged in feedstock cultivation. Nonetheless, biodiesel production faces a great challenge due to the substantial amounts of impure crude glycerol produced as a by-product. With recent expansions in global biodiesel production, there has been an excess of crude glycerol which is typically classified as waste. Consequently, various approaches for its utilization are currently under investigation [3]. Currently, 1 ton of glycerol is generated for every 10 tons of biodiesel produced. According to the IEA, the transport biofuel forecast predicts an increase from 37.1 to 44.1 billion L from 2019 to 2024.

Glycerol is considered a “building block molecule” in the biorefinery field because it participates in various types of chemical reactions, and thus has potential for conversion into several value-added chemicals, such as 1,2-propanediol, acetol, 1,3-propanediol, or acrolein, among others [4,5,6,7]. In this context, some aqueous processes such as aqueous-phase reforming (APR) and aqueous-phase hydrogenolysis (APH) are being investigated to convert glycerol into value-added chemicals. APR was developed by Dumesic and his coworkers in 2002 [8]. This process enables the conversion of aqueous solutions into gases, especially hydrogen and alkanes. The review by Coronado et al. [9] identified the development of active and stable catalysts and the optimization of operating conditions as the main challenges for APR technology. Gas production is favored at high temperatures and high ratios of catalyst weight/organic feed [10,11]. Moreover, the active phase of the catalyst significantly influences gas production [12]. The APR of waste streams such as cheese whey, brewery wastewater, and fish-canning industry effluents has also been investigated [13,14,15].

As generally studied, the aqueous-phase hydrogenolysis process (APH) of glycerol involves an external hydrogen supply. However, the process examined in this study explores APH without an external hydrogen source. A temperature and pressure of 227 °C and 34 bar were used, aiming at improving the production of value-added chemicals. In these conditions, glycerol is converted simultaneously to gaseous and liquid products, and the produced hydrogen further participates in hydrogenolysis reactions to generate chemicals such as 1,2-propanediol (1,2-PDO). As such, the reaction pathways to convert glycerol include reforming reactions to generate gases [12] and reactions in the liquid phase. Many researchers have studied glycerol conversion in the aqueous phase and generally acknowledge the participation of the acid sites on the support, where glycerol is firstly dehydrated to produce acetol, and that of the metal active sites, where acetol is hydrogenated to generate 1,2-PDO [16,17].

The catalyst exerts a relevant role in aqueous processes and, in this study, Ni/CeO_2_ was selected as the catalyst. For instance, ceria has been used as an active phase [18,19], as a promoter [20,21], and as support [22,23] in catalytic systems in a wide variety of processes. One of the advantages of ceria is its ability to store and release oxygen, favored by the presence of oxygen vacancy sites. Ceria has also been demonstrated to be resistant to water [18], which is relevant in aqueous-phase processes. Ni/CeO_2_ catalysts have been employed in the APR of n-butanol and ethanol [24,25,26].

Manfro et al. [23] studied Ni/CeO_2_ catalysts prepared by several methods in the APR of glycerol at 523 and 543 K in a batch system, focusing on gas production. Lee et al. [27] investigated nickel-supported catalysts for glycerol APR, concluding that glycerol conversion to gas is significantly influenced by the catalyst and operating conditions. Ni/CeO_2_ showed the lowest glycerol conversion to gas and the highest selectivity to CO. In recent work, Ni/Ce_x_-Zr_1−x_-O_2_ catalysts were also tested for alkane production [28]. However, liquid products from glycerol in pressure aqueous processing have not been reported using Ni/CeO_2_ catalysts.

The present work studies the effect of the synthesis conditions of Ni/CeO_2_ catalysts on the APH of glycerol without a hydrogen supply. In particular, CeO_2_ support calcination temperatures (500 and 700 °C) and Ni/CeO_2_ catalyst calcination temperatures (600, 700, and 800 °C) were assessed; in these assessments, the Ni content varied between 10 to 20 wt.%. All catalysts were prepared by impregnation. Characterization techniques such as N_2_ adsorption, X-ray diffraction, H_2_-TPR, and TPD-NH_3_, among others, were employed to explain the results obtained. Furthermore, both gaseous and liquid products were analyzed by gas chromatography.

## 2. Results and Discussion

### 2.1. Catalyst Characterization Results before the Reaction

#### 2.1.1. Chemical Analysis and Textural Properties

**Figure 1:**
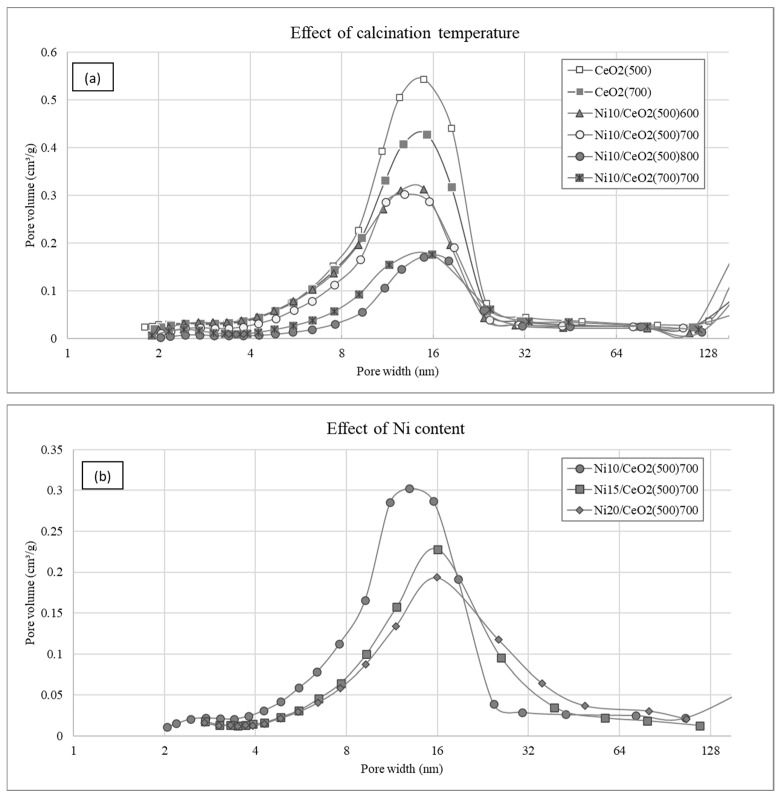
Effect of (**a**) calcination temperature and (**b**) Ni content on pore volume distribution.

The chemical analysis and textural properties results are shown in Table 1. The nomenclature of the catalysts is explained in Section 3.1. The incorporation of the Ni into the catalysts is confirmed via ICP-OES analysis. The effect of the calcination temperature on the textural properties of the CeO_2_ supports and the impregnated catalysts heavily affect the textural properties (see Figure 1) [19]. An increase in such temperatures leads to a significant decrease in the surface area and the formation of higher-diameter and low-volume pores (see Figure S1 for further information). Low metallic dispersion values (D_exp_ ranging from 1.13 to 2.72%), were determined experimentally in all the catalysts, as reported in previous work related to Ni/CeO_2_ catalysts [29].

An increase in the nickel content leads to a reduction in the specific surface area and larger pore diameters, while the pore volumes remain largely unaffected. These outcomes are consistent with prior investigations conducted by Manfro et al. [23], where the catalysts exhibited analogous textural characteristics, and an identical impact on surface area was noted after the Ni impregnation of the CeO_2_ support.

#### 2.1.2. X-ray Diffraction

**Figure 2:**
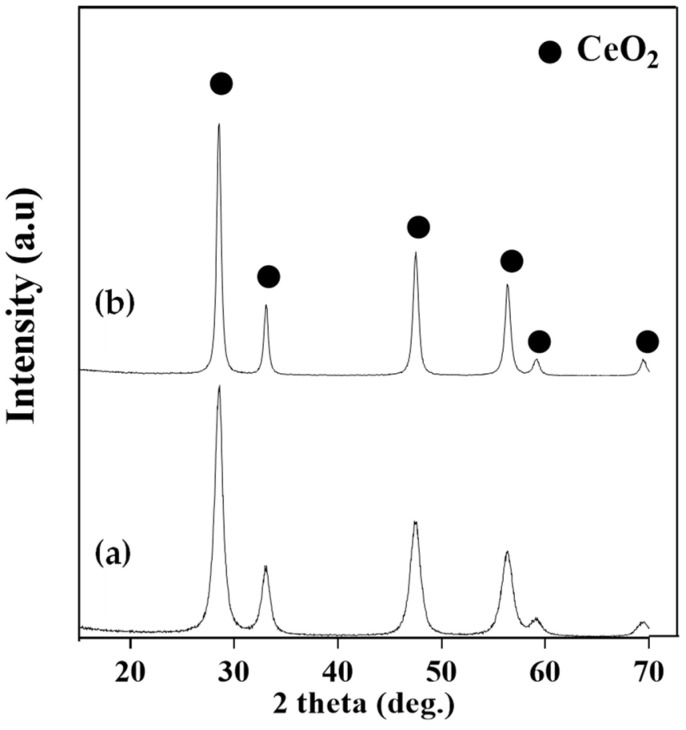
XRD patterns of CeO_2_ supports: (**a**) CeO_2_(500) and (**b**) CeO_2_(700).

**Figure 3:**
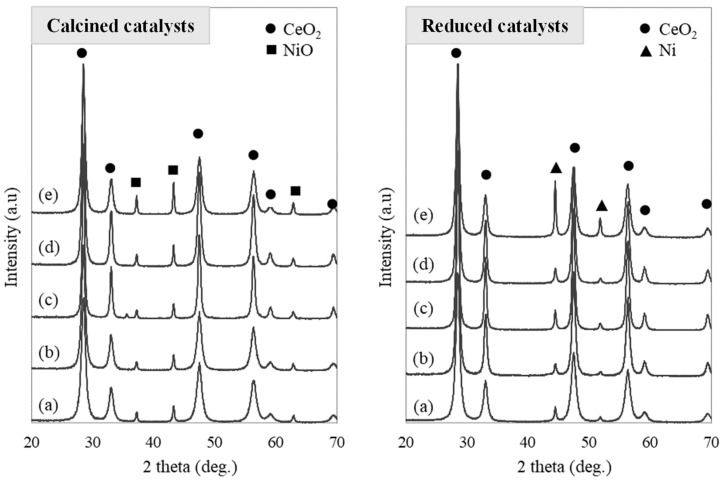
XRD diffractograms of calcined (**left**) and reduced (**right**) catalysts: (**a**) Ni10/CeO_2_(500)600, (**b**) Ni10/CeO_2_(500)700, (**c**) Ni10/CeO_2_(500)800, (**d**) Ni10/CeO_2_(700)700, and (**e**) Ni20/CeO_2_(500)700.

The XRD diffractograms of the catalytic supports calcined at 500 and 700 °C are shown in Figure 2, while Figure 3 shows the XRD diffractograms for the calcined and reduced catalysts. Further details about Ni and CeO_2_ crystallite sizes for the reduced catalysts can be found in the Supplementary Materials (see Table S1). All the calcined catalysts show signals relating to NiO and CeO_2_ crystalline phases. The peaks of the crystalline phase of CeO_2_ are identified at 28, 33, 47, 59, and 69° (JCPDS card No. 043-1002). The increase in the calcination temperature of the support generates sharper signals in the XRD pattern, which correspond to higher crystal sizes. This tendency of the crystallite size to increase with the increase in the calcination temperature of CeO_2_ was also observed by Smith et al. [19].

High-intensity NiO signals are observed at 37, 43, and 63° (JCPDS card No. 047-1049) for the catalyst Ni20/CeO_2_(500)700. The highest values of CeO_2_ crystallite size were obtained in the calcined catalysts Ni10/CeO_2_(700)700 and Ni10/CeO_2_(500)800, being 15.4 and 18.5 nm, respectively. Therefore, a temperature increase, both in the preparation of the catalyst support and in the calcination of the catalyst, significantly influences their crystalline and porous structures. The highest NiO crystallite sizes were observed for the catalyst with the highest Ni loads, Ni20/CeO_2_(500)700. This phenomenon was also reported in the previous work by Manfro et al. [23] when the impregnated catalyst was calcined at 500 °C.

The reduction temperature employed for each specific catalyst was selected based on the results from the H_2_-TPR analysis (see Section 2.1.3). The same temperatures were used when performing the in situ reduction of the catalysts, before the APH tests. All the reduced catalysts show signals of Ni and CeO_2_ crystalline phases. Ni signals appeared at 44 and 52° (JCPDS card No. 004-0850). No signals of NiO were shown, indicating the complete reduction of NiO to Ni. An increase in Ni intensity peaks was observed for the Ni20/CeO_2_(500)700 catalyst.

#### 2.1.3. H_2_-TPR Results

**Figure 4:**
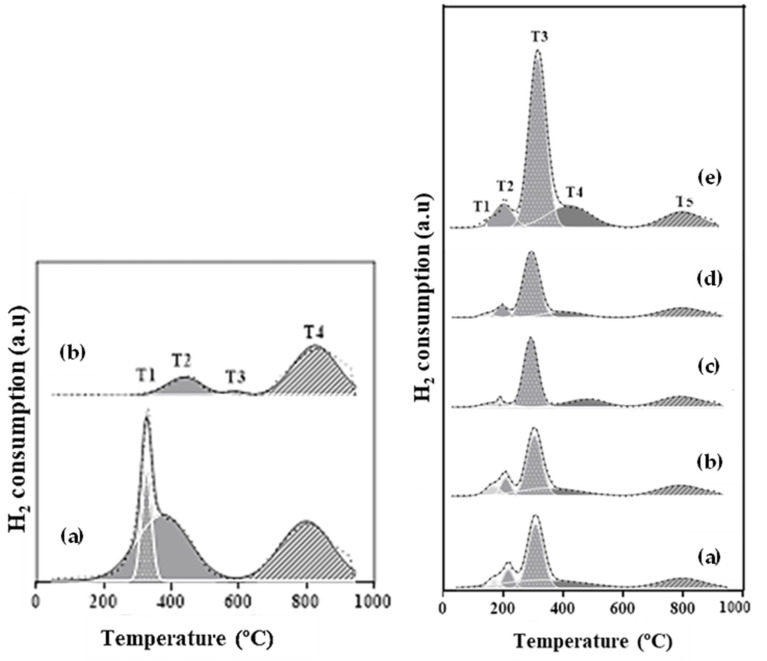
H_2_-TPR profiles of CeO_2_ supports (**left**): (**a**) CeO_2_(500) and (**b**) CeO_2_(700); H_2_-TPR profiles of Ni/CeO_2_ catalysts (**right**): (**a**) Ni10/CeO_2_(500)600, (**b**) Ni10/CeO_2_(500)700, (**c**) Ni10/CeO_2_(500)800, (**d**) Ni10/CeO_2_(700)700, and (**e**) Ni20/CeO_2_(500)700.

Figure 4 shows the H_2_-TPR profiles of the CeO_2_ supports and the catalysts. The specific temperature values for each peak are detailed in Table 2. For the CeO_2_ supports, two temperature regions are observed, from 200 to 600 °C and from 600 to 950 °C. The CeO_2_ support calcined at 500 °C presents three peaks at 326, 374, and 800 °C, while the support calcined at 700 °C has two main peaks at 438 and 827 °C. According to the literature, the H_2_ consumption in the first region might correspond to the reduction of superficial CeO_2_ and/or oxygen vacancy defects (OVD), which are easier to reduce, while in the second region, the H_2_ consumption is attributed to bulk CeO_2_. These two regions were also found in other studies [22,30,31]. The lower temperature peak appears to be influenced by the preparation procedure of the CeO_2_ support, as well as by the calcination temperature. The first peak of the CeO_2_ support calcined at 500 °C, occurring at 326 °C, which may be characteristic of CeO_2_ with a high surface area.

In the case of the catalysts, five peaks were identified after the profile deconvolution (T1 to T5). Peak T5 is in the high-temperature region, from 798 to 823 °C. The rest of the peaks are found at lower temperatures. Peaks T1 and T2, at temperatures ranging from 160 to 227 °C, correspond to the reduction of oxygen vacancy defects (OVD) in the CeO_2_ structure [30,32]. The works of Mamontov et al. [33,34] indicate that OVD are due to the presence of Frenkel-type defects in the ideal fluorite structure of CeO_2_. These defects consist of interstitial oxygen ions shifted from octahedral sites. OVD formation can occur by thermal treatments with air and/or hydrogen [35], or superficial chemical treatments with supported metals [36]. Tang et al. [30] also suggested that oxygen vacancies are generated in CeO_2_ supports due to ceria lattice expansion during the thermal treatment. OVD in Ni/CeO_2_ catalysts are caused by the dissolution of nickel into the ceria matrix. This effect was also observed in the work of Tang et al., which hinted that the Ni addition could promote the formation of oxygen vacancies, this being a possible explanation for the high amount of OVD in the Ni20/CeO_2_(500)700 catalyst. Furthermore, these vacancies could act as active sites for the adsorption and activation of reactant molecules, improving their catalytic activity and selectivity [37].

In the present work, the Ni10/CeO_2_(500)700 catalyst presented the smallest CeO_2_ particle size in the calcined catalyst and a low T4 temperature value. Furthermore, the T3 peaks, at temperatures from 308 to 341 °C, are associated with the reduction of superficial NiO or NiO in weak interaction with CeO_2_ [22,30]. Peak T4, at temperatures from 335 to 491 °C, is associated with the reduction of NiO with strong interactions with the support and superficial CeO_2_. Peak T5 corresponds to the reduction of bulk CeO_2_ [30].

#### 2.1.4. NH_3_-TPD Results

**Figure 5:**
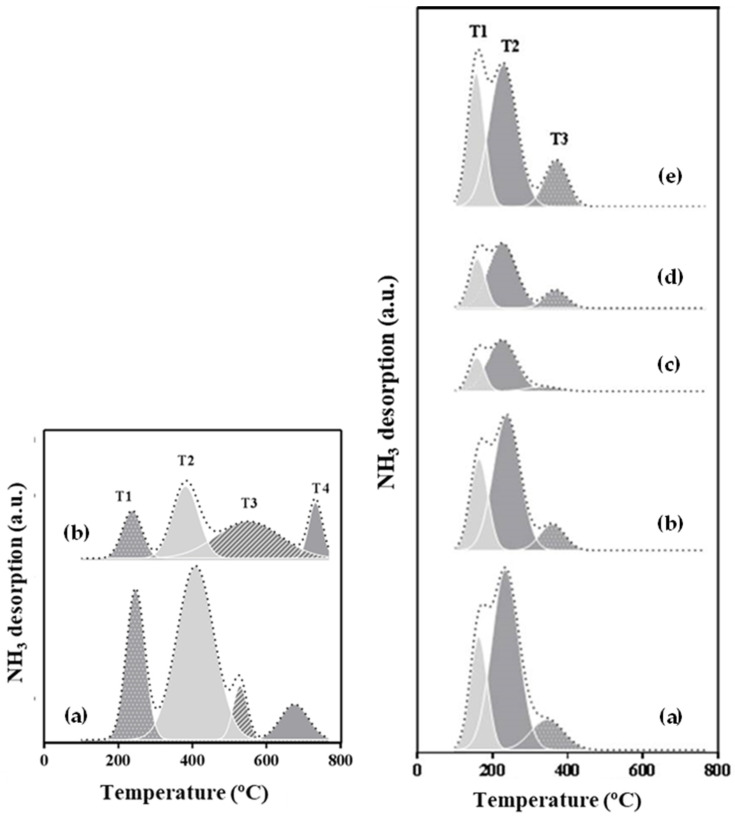
NH_3_-TPD profiles of CeO_2_ supports (**left**): (**a**) CeO_2_(500) and (**b**) CeO_2_(700); NH_3_-TPD profiles of Ni/CeO_2_ catalysts (**right**): (**a**) Ni10/CeO_2_(500)600, (**b**) Ni10/CeO_2_(500)700, (**c**) Ni10/CeO_2_(500)800, (**d**) Ni10/CeO_2_(700)700, and (**e**) Ni20/CeO_2_(500)700.

The results of the NH_3_-TPD analysis are shown in Figure 5 and Table 3. The temperatures of all the peaks are given, along with the strength distributions of the acid sites as follows: low, low-medium, medium, and high. Total acidity is presented, expressed as µmol NH_3_/g and µmol NH_3_/m^2^. The last value is calculated as the total acidity in µmol NH_3_/g divided by the surface area.

Two regions can be observed in the CeO_2_ support profile, the first from 100 to 500 °C and the second from 500 to 800 °C. The second region is associated with medium to strong acid sites. The CeO_2_ support calcined at 500 °C presents four peaks at 245, 413, 528, and 675 °C, with a total acidity of 64 µmol NH_3_/g. Although the CeO_2_ supports are less acidic materials, they show a higher distribution of medium to high-strength acid sites than the Ni/CeO_2_ catalysts. This value is similar to that determined by Yang et al. [38]. The CeO_2_ support calcined at 700 °C displays four peaks at 241, 372, 551, and 740 °C, with a total acidity of 18 µmol NH_3_/g. A clear decrease in total acidity when the calcination temperature of the support increases is observed. The values of total acidity per surface area are 0.65 and 0.30 µmol NH_3_/m^2^ for the supports calcined at 500 and 700 °C, respectively.

Three peaks appear after the profiles’ deconvolution (T1 to T3) of the Ni/CeO_2_ catalysts. These peaks are in the first region, at temperatures lower than 500 °C. The first peak, T1, at temperatures from 145 to 163 °C, is associated with low-strength acid sites. The second peak, T2, with a maximum at temperatures from 219 to 238 °C, is related to low-medium strength acid sites, and the third peak, T3, with a maximum at temperatures from 337 to 367 °C, is associated with medium-strength acid sites. All the catalysts display a significant increase in the total acidity expressed as µmol NH_3_/m^2^ compared to the supports. A notable increase is observed from 2.80 to 3.79 µmol NH_3_/m^2^ for the catalysts with 10 and 20 wt.% of Ni load, respectively. The Ni10/CeO_2_(500)700, Ni10/CeO_2_(700)700, and Ni20/CeO_2_(500)700 catalysts have the highest proportions of medium acid sites and the highest values of the maximum of T3. The formation of acid sites with the incorporation of Ni was also found by other researchers using different supports [38]. Furthermore, the introduction of nickel could enhance the creation of new OVD, which are also related to the acidity properties of the catalyst (see Figure S4). It must be noted that some acid sites might disappear when reducing the catalyst since Lewis acid sites are destroyed. However, CeO_2_ supports acidity, and the OVD present in the CeO_2_ lattice could particularly contribute to keeping the catalysts’ acidic properties [39,40].

**Figure 6:**
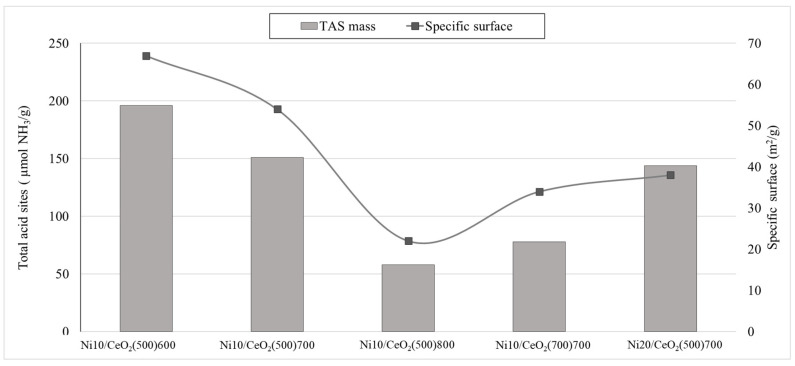
Effect of specific surface on the catalytic acidity (expressed on a mass basis).

The increase in the calcination temperature of the catalysts causes a decrease in the total acidity expressed as µmol NH_3_/g, with values from 58 to 196 µmol NH_3_/g for the catalysts Ni10/CeO_2_(500)800 and Ni10/CeO_2_(500)600, respectively. As a consequence of the effect of the specific surface on the catalyst acidity (see Figure 6), the acidity was also expressed as µmol NH_3_/m^2^ (TAS_surface_), aiming at showing a surface-based parameter. Thus, the support calcination temperature effect is corroborated when the Ni10/CeO_2_(500)700 and Ni10/CeO_2_(700)700 catalysts are compared, showing a clear decrease in the total acidity (ranging from 2.80 to 2.29 µmol NH_3_/m^2^).

### 2.2. Catalytic Activity

#### 2.2.1. Global Activity Results

The activity results of the catalysts are shown in Table 4. No significant activity was detected for the CeO_2_(500) and CeO_2_(700) supports, as the glycerol conversion values achieved were lower than 5%. Additionally, the carbon deficit values are equal to or smaller than 6.5%, which is an acceptable value for the reliability of the experiments according to the literature (see Section 3.3) [11,41].

The observed glycerol conversion values ranged from 17.1 to 29.0%, the latter achieved by the catalyst Ni10/CeO_2_(500)700. Carbon yield to liquids (CYliq) values varied from 12.6 to 24.0%. The Ni10/CeO_2_(700)700, Ni10/CeO_2_(500)700, and Ni20/CeO_2_(500)700 catalysts presented the highest glycerol conversion and CYliq values. Gas production was not remarkable, yielding values lower than 2% in all the experiments. Such low gas production differs significantly from previous results obtained in similar processes with Ni–Al coprecipitated catalysts and the same operating conditions, in which the observed carbon yield to gas (CYgas) and CYliq values were around 17%, and 27%, respectively [11]. The generation of metallic sites following the addition of metals (Ni, Pt) has been identified as a crucial factor in producing gaseous products, particularly hydrogen (H_2_), in various studies [11,42,43]. Specifically, when Ni was incorporated into a CeO_2_ support (Ni/CeO_2_) in these studies, the formation of significant amounts of gaseous products was not observed. Instead, the catalyst predominantly promotes the production of liquid products. This behavior is strongly influenced by the acidity of the catalyst, which plays a pivotal role in dictating product distribution.

**Figure 7:**
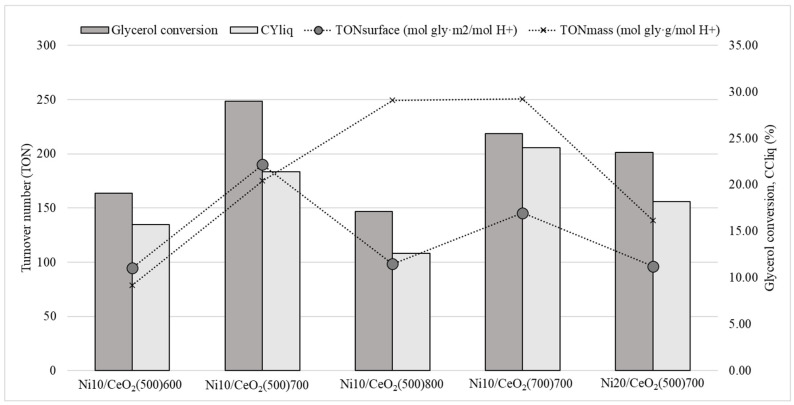
Catalytic activity results.

The acidity of the catalysts has been reported to be relevant in the pathway of glycerol conversion by APR and hydrogenolysis [16]. The nickel addition to CeO_2_ supports leads to the generation of new active sites and a significantly more active catalyst. Nevertheless, when contrasting the activity of catalysts with identical nickel content, it becomes evident that additional parameters exert an influence on their activity, regardless of the quantity of impregnated metal. Specifically, investigations have been conducted to examine the impact of textural properties on the formation of acidic sites. To evaluate the influence of the acid sites and the specific surface on their catalytic activity, the turnover number (TON) was calculated. Due to the low metallic dispersion values in all the catalysts (especially those with lower surface area), and the predominant route towards liquid products, the TON was calculated considering the acid sites as active sites of the catalyst. This calculation was conducted from two different perspectives: (1) the total amount of acid sites per specific surface (i.e., TON_surface_, see Equation (5)) and (2) the total amount of acid sites per gram of catalyst (i.e., TON_mass_, see Equation (6)). According to Figure 7**,** the trend followed by the TON_mass_ presents a clear discrepancy with the overall catalytic activity results, especially in the case of the catalyst with the lowest specific surface (i.e., Ni10CeO_2_(500)800). This supports the hypothesis that the new acid sites created by the addition of Ni to the CeO_2_ not only depend on the metal content but also on the textural properties of the catalyst, showing a direct effect on the CYliq values (see Figure 7).

**Figure 8:**
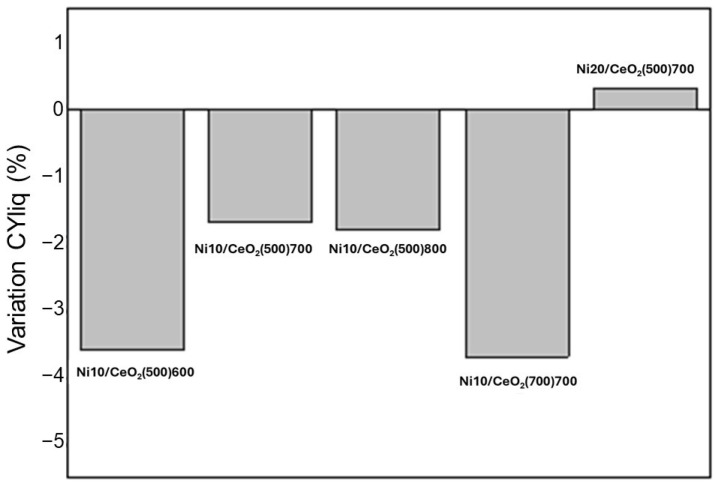
Variation of CCliq (%) from the third to the first hour of reaction.

Catalytic activity was evaluated throughout the 3 h reaction. No significant variations in the catalytic activity were observed in this period (see Figure 8). Only the catalysts Ni10/CeO_2_(500)600 and Ni10/CeO_2_(700)700 showed variations close to 4%.

#### 2.2.2. Products Distribution

**Figure 9:**
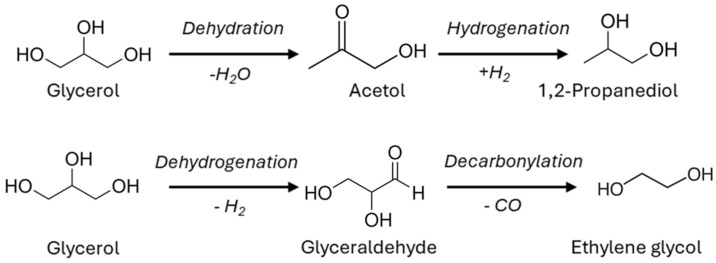
Reaction pathway: glycerol conversion to 1,2-PDO and EG.

The detailed results of the yields of the liquid products 1,2-propanediol (1,2-PDO), acetol, ethylene glycol (EG), and ethanol (EtOH) obtained for all the studied catalysts are detailed in Table 4. The main reaction pathways are depicted in Figure 9. Yield values (Equation (8)) are determined by the carbon yield to liquids, CYliq (Equation (2)), and the carbon selectivity of liquid products S_A_ (Equation (9)). While the value-added liquids with the highest yields were 1,2-PDO and acetol, EG and EtOH were also produced in significant amounts in some catalysts.

The highest acetol yield, 0.1136 g/g glycerol, was obtained with the Ni10/CeO_2_(700)700 catalyst. The highest 1,2-PDO yields, around 0.06 g/g glycerol, were obtained with the Ni20/CeO_2_(500)700 and Ni10/CeO_2_(500)700 catalysts. Furthermore, the highest EG yield, with values around 0.014 g/g glycerol, was obtained with the Ni20/CeO_2_(500)700 and Ni10/CeO_2_(500)700 catalysts. Finally, the highest EtOH yields with a value of circa 0.02 g/g glycerol were achieved with the Ni10/CeO_2_(700)700, Ni20/CeO_2_(500)700, and Ni10/CeO_2_(500)700 catalysts.

As previously mentioned, the Ni20/CeO_2_(500)700 catalyst showed the highest yields to 1,2-PDO and EG, with 0.061 g/g glycerol and 0.015 g/g glycerol, respectively. This is related to its higher Ni content, leading to a higher generation of hydrogen in the gas phase and consequently enhancing the hydrogenation of the acetol to 1,2-PDO, and also C–C bond breakage reactions.

**Figure 10:**
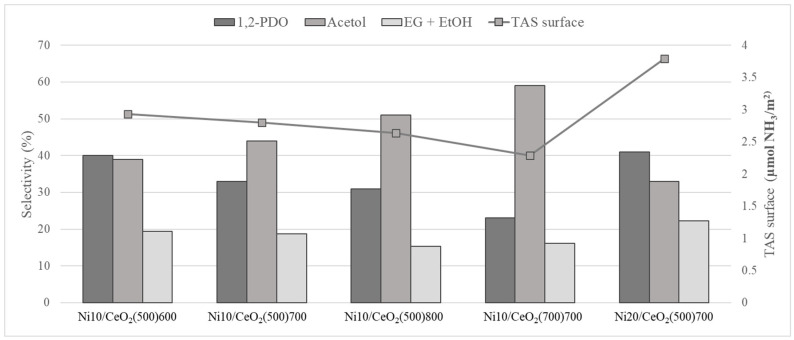
Acidity effects on the selectivity towards main products.

Furthermore, the total acidity seems to have a direct effect on the reaction pathway, enhancing the selectivity towards 1,2-PDO and other products, in which C–C bond breakage takes place (i.e., EG + EtOH) (see Figure 10).

These results differ from previous works catalyzed by different Ni-based catalysts. For instance, the acetol and 1,2-PDO yields obtained in the work of García et al. [11] with the coprecipitated Ni–Al catalyst at the same operating conditions (34 bar absolute pressure, 500 K, 5 wt.% glycerol, 1 g of glycerol and 1 mL/min of liquid feeding rate, W/m_glycerol_ = 20 g catalyst min/g glycerol) were of 0.033 g/g glycerol and 0.098 g/g glycerol, respectively. These results indicate that acetol production with the Ni10/CeO_2_(700)700 and Ni10/CeO_2_(500)700 catalysts is more than doubled in comparison with the Ni–Al coprecipitated catalyst. Therefore, it confirms the higher tendency of these Ni/CeO_2_ catalysts to produce liquids compared to other catalytic systems, which favor the production of gaseous products.

The global results of carbon selectivity to liquid products are presented in Table 4. The results show that the main liquid products generated are 1,2-PDO, acetol, EtOH, and EG, and their production is aligned with the acidity results presented previously. The values of carbon selectivity to 1,2-PDO and acetol together are higher than 73% for all the experiments. This shows that the most favored reaction route is the dehydration of glycerol to produce acetol, followed by the hydrogenation of acetol to generate 1,2-PDO [10].

The carbon selectivity to 1,2-PDO varied from 23 to 41%. The catalyst with the lowest carbon selectivity to 1,2-PDO is Ni10/CeO_2_(700)700, which is the catalyst with the lowest total acidity. On the other hand, the Ni10/CeO_2_(500)600 and Ni20/CeO_2_(500)700 catalysts present values of carbon selectivity to 1,2-PDO close to 40%.

Regarding acetol production, the carbon selectivity of this product varied from 33 to 59%. The Ni20/CeO_2_(500)700 catalyst presents the lowest value of carbon selectivity to acetol and the highest carbon selectivity to 1,2-PDO, again suggesting the positive effect of a higher Ni content and acidity on the hydrogenation step to convert acetol in 1,2-PDO. Conversely, the Ni10/CeO_2_(700)700 catalyst shows the highest carbon selectivity to acetol, at 59%, and it is the catalyst with the lowest carbon selectivity to 1,2-PDO.

The carbon selectivity to EtOH and EG varied from 9.3 to 13.8%, and from 4.5 to 8.4%, respectively. The lowest selectivity values of both compounds were obtained by the lowest acid catalysts (i.e., Ni10/CeO_2_(500)800 and Ni10/CeO_2_(700)700, respectively), in which dehydration reactions are less promoted. In opposition, the Ni20/CeO_2_(500)700 catalyst presents the highest carbon selectivity to EtOH and EG, in which C–C bond breakage reactions are also enhanced by higher Ni content.

These results indicate that the most active catalysts in this study, with high glycerol conversion, are Ni10/CeO_2_(500)700, Ni10/CeO_2_(700)700, and Ni20/CeO_2_(500)700. These catalysts have high proportions of medium-strength acid sites. Accordingly, the carbon selectivity to liquid products could be related to the total acidity expressed as µmol NH_3_/m^2^. The highest carbon selectivity to 1,2-PDO and EG was obtained with the Ni20/CeO_2_(500)700 catalyst, which has the highest total acidity with a value of 3.79 µmol NH_3_/m^2^. The highest carbon selectivity to acetol was obtained with the Ni10/CeO_2_(700)700 catalyst, which has the lowest total acidity, 2.29 µmol NH_3_/m^2^.

No significant temporal variations were observed in terms of the products’ selectivity (see Figure S6 for further information). In all cases, lower values of selectivity to acetol were observed. A higher selectivity to 1,2-PDO was stated for the catalyst Ni20/CeO2(500)700, probably due to a higher generation of H_2_ in the reaction medium.

### 2.3. Catalyst Characterization Results after the Reaction

#### 2.3.1. X-ray Diffraction

**Figure 11:**
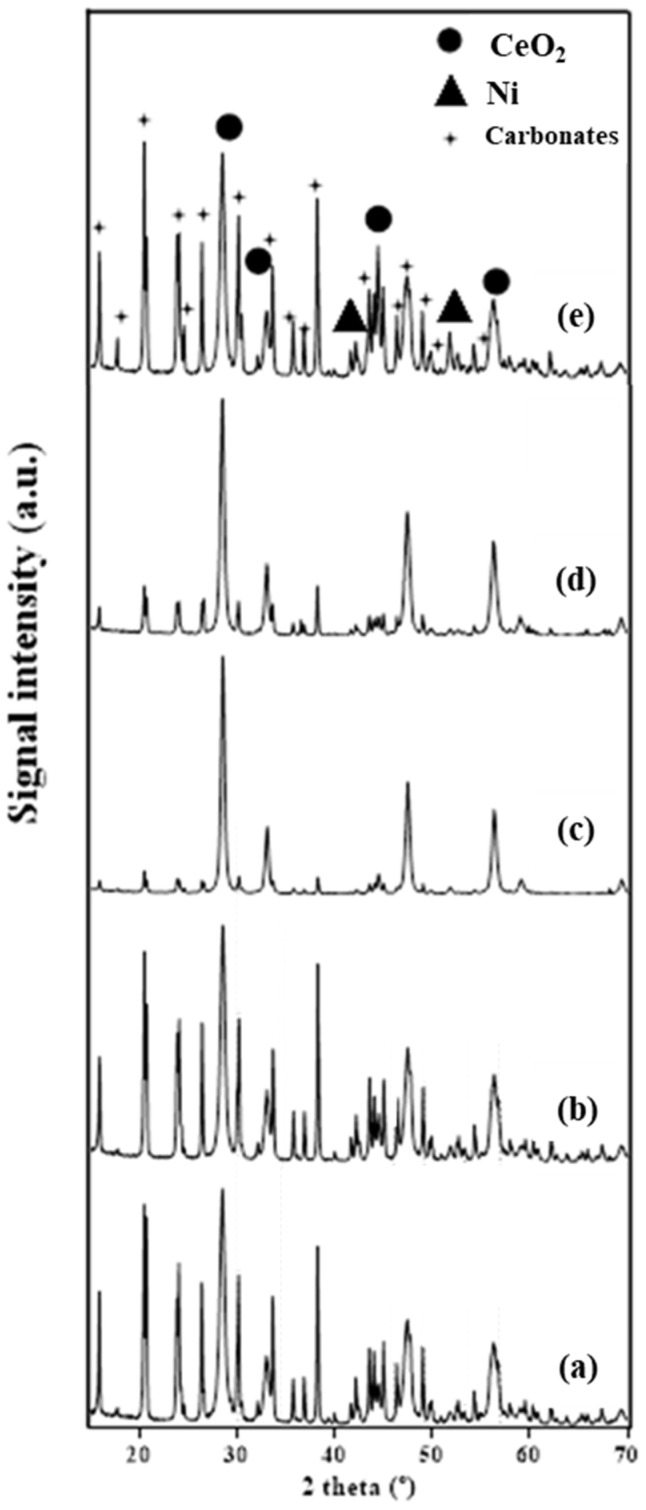
XRD diffractograms of spent Ni/CeO_2_ catalysts: (**a**) Ni10/CeO_2_(500)600, (**b**) Ni10/CeO_2_(500)700, (**c**) Ni10/CeO_2_(500)800, (**d**) Ni10/CeO_2_(700)700, and (**e**) Ni20/CeO_2_(500)700.

The spent catalysts were characterized after the experiments by XRD (see Figure 11). All the samples show Ni and CeO_2_ phases, and crystalline phases associated with cerium carbonates Ce_2_O(CO_3_)_2_H_2_O (JCPDS card No. 044-0617) and Ce(OH)CO_3_ (JCPDS card No. 032-0189). CO_2_ is a by-product of the APH process, thus leading to the formation of CO_3_^2−^ions in aqueous media. Cerium-derived ions can exist [Ce(H_2_O)_n_^3+^] in aqueous media [44,45]. Subsequently, these ions can be transformed into other species, such as Ce(OH)(H_2_O)_n−1_^2+^, to react with the carbonates. Lu and Wang determined that the phase transformation from cerium carbonate hydroxide to cerium oxide appears at around 250 °C [46]. The experiments were conducted at 227 °C; consequently, this cerium carbonate hydroxide phase could be present.

Ni10/CeO_2_(700)700 and Ni10/CeO_2_(500)800 catalysts showed a low intensity of the cerium carbonate crystalline phases. This fact could be related to the higher calcination temperatures, consequently showing the lowest surface areas. However, the existence of greater or smaller amounts of cerium carbonate crystalline phases does not appear to be related to catalyst activity in the 3 h experiments, according to the results in Table 4.

#### 2.3.2. SEM Microscopy

**Figure 12:**
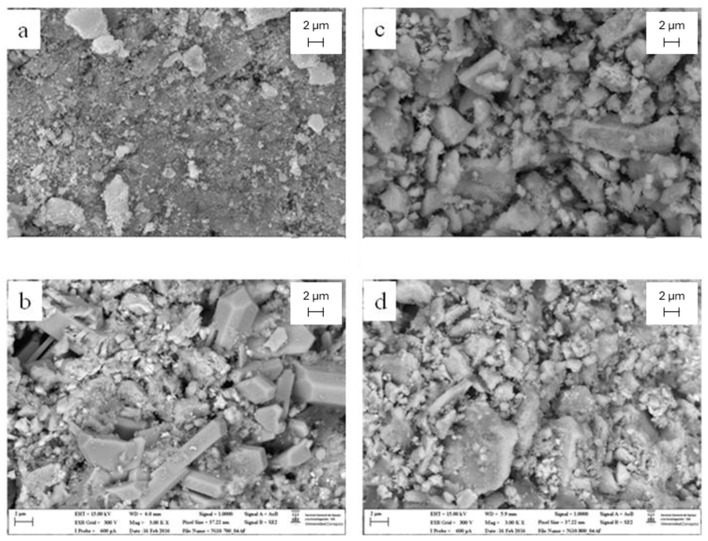
SEM images: (**a**) calcined Ni10/CeO_2_(500)700, (**b**) used after reaction Ni10/CeO_2_(500)700, (**c**) calcined Ni10/CeO_2_(500)800, and (**d**) used after reaction Ni10/CeO_2_(500)800.

Figure 12a,b show SEM images corresponding to the Ni10/CeO_2_(500)700 catalyst calcined and used after the reaction, respectively. The modification of the catalyst surface after the chemical reaction is clear, with the presence of big crystals in the shape of nanorods between 2 and 6 µm in length. These big crystals are likely the cerium carbonate crystalline phases detected in the XRD analysis and corroborated by EDX.

Figure 12c,d show SEM images corresponding to the Ni10/CeO_2_(500)800 catalyst calcined and used after the reaction, respectively. Scarcely any modification of the catalyst surface is observed for this catalyst. This is corroborated by the lowest intensity in the XRD patterns of cerium carbonate crystalline phases, as seen in Figure 9.

#### 2.3.3. Elemental Analysis

The carbon content of the catalysts after the reaction was determined and is presented in Table 5. Coke formation is significantly influenced by the operating conditions; in particular, its formation has been reported at 250 °C [23]. At the operating conditions employed in the present work (227 °C and 34 bar), coke formation is minimal. Moreover, the presence of CeO_2_ in the catalyst also hinders coke formation due to its oxygen storage capacity [47].

Ni and Ce contents in the liquid stream were also determined by ICP-OES to quantify the metal leaching during the reaction (Table 5). The detected amounts were not remarkable; however, they could be related to the activity variations described in Figure 8, especially in the case of Ni10/CeO_2_(500)600 and Ni10/CeO_2_(700)700.

The Ni10/CeO_2_(700)700 and Ni10/CeO_2_(500)800 catalysts have the lowest carbon content. These are the catalysts with low intensities of cerium carbonate crystalline phases revealed by the XRD analysis after the reaction (Figure 11). Therefore, the carbon content in spent catalysts could be related to cerium carbonates.

## 3. Materials and Methods

### 3.1. Catalyst Preparation

The Ni/CeO_2_ catalysts were prepared via incipient wetness impregnation. Ceria supports were prepared previously by Ce(NO_3_)_3_·6H_2_O calcination under an air flow (100 cm^3^ STP/min) at the final calcination temperature of the support, 500 or 700 °C, for 3 h. The support was then impregnated with a known volume of Ni(NO_3_)_2_·6H_2_O solution. Catalysts with 10 and 20 wt.% Ni load were prepared. Ce(NO_3_)_3_·6H_2_O and Ni(NO_3_)_2_·6H_2_O analytical grade from Sigma Aldrich and milli-Q water were used for this process.

The hydrated catalyst precursors were dried at 105 °C and then calcined under 95 cm^3^ STP/min flow of synthetic air at the final calcination temperature of the catalyst for three hours. Finally, the catalysts were ground and sieved to a particle size between 160 and 315 µm.

The nomenclature of the catalysts is as follows: NiX/CeO_2_(T1)T2, where X indicates the theoretical Ni load, T1 is the calcination temperature of the support, and T2 is the calcination temperature of the catalyst.

Catalysts were activated by reduction at 500 or 550 °C (100 cm^3^ STP/min hydrogen flow) for 1 h, according to the H_2_-TPR results. Table 6 presents the catalysts and the main preparation conditions.

### 3.2. Catalyst Characterization

The specific surface area and pore distribution of the catalysts and the supports were determined by N_2_ physisorption at −195.65 °C with a TriStar 3000 V6.08 instrument (Micromeritics Instrument Corporation, Norcross, GA, USA). The specific surface area was determined following the multi-point BET procedure and the pore-size distribution with the BJH method, using the adsorption branch of the isotherms. The pore thickness was determined using the Harkins and Jura equation (t = [13.99/(0.034 − log(P/Po))]^0.5^).

The XRD analyses were performed at room temperature on the calcined, reduced, and used samples using an X-ray diffractometer (RIGAKU, D/max 2500, Rigaku Corporation, Tokyo, Japan) equipped with a rotatory anode. The diffractometer operates at 40 kV and 80 mA, with a Cu anode and a graphite monochromator, using Cu Kα radiation. The analyses were conducted under the following conditions: 2θ within 15 to 70°, with a step of 0.03°/s. The JCPDS–International Centre for Diffraction Data-2000 was used.

H_2_-TPR (temperature programmed reduction) was employed to study the reducibility of the nickel and cerium oxide species. The analyses were carried out using an AUTOCHEM II 2920 instrument (Micromeritics Instrument Corporation, Norcross, GA, USA) equipped with a thermal conductivity detector. The samples were dried in an Ar atmosphere (50 STP cm^3^/min) until 110 °C (10 °C/min) for 30 min. The subsequent sample reduction was performed in a 10 vol.% H_2_/Ar atmosphere (50 STP cm^3^/min) until 950 °C (10 °C/min).

NH_3_-TPD (temperature-programmed desorption of ammonia) was performed using the AUTOCHEM II 2920 instrument (Micromeritics Instrument Corporation, Norcross, GA, USA) equipped with a thermal conductivity detector. The calcined catalysts were dried first in an He stream at 450 °C for 1 h and then cooled at 100 °C in the He stream. The ammonia adsorption was carried out at 100 °C using a mixture of 0.5% NH_3_/He with a flow rate of 50 STP cm^3^/min for 1 h. After the adsorption, the samples were purged with flowing He at 100 °C for 1 h to remove the physisorbed ammonia. The desorption of the chemisorbed ammonia was measured by heating the samples to 770 °C at a rate of 10 °C/min (total flow 30 STP cm^3^/min).

*Inductively coupled plasma optical emission spectrometry* (*ICP-OES*), using Thermo Elemental IRIS Intrepid equipment, was conducted to corroborate the catalysts’ Ni content in the calcined samples.

*Elemental analysis* was performed to quantify the carbon content in the spent catalysts, determined by a Leco CHN628 elemental analyzer equipped with NDIR infrared cells.

*H*_2_-*Chemisorption* was performed with a Micromeritics Autochem II at 30 °C using a dynamic method with hydrogen pulses. The catalysts were previously reduced in situ at the same temperature as that employed in the catalytic tests. 10% H_2_ diluted in Ar was used, and a flow rate of 50 STP cm^3^/min.

### 3.3. Catalyst Performance

The catalysts’ performance was tested experimentally in a small bench-scale continuous unit designed and developed by PID Eng&Tech (Madrid, Spain). A diagram of the installation can be found elsewhere [10]. The gas stream was analyzed and quantified online by an Agilent 490 Micro-GC analyzer, equipped with thermal conductivity detectors (TCD) and calibrated before each experiment.

Catalysts were tested for 3 h, at 227 °C and 34 bar of absolute pressure, using a 5 wt.% glycerol aqueous solution as the feed, a liquid flow of 1 mL/min, and a mass of catalyst/glycerol mass flow rate ratio (W/m_glycerol_) of 20 g catalyst min/g glycerol. Before the reaction, the catalyst was activated by an in situ reduction step.

The liquid product stream, consisting of 1,2-PDO, acetol, ethanol (EtOH), ethylene glycol (EG), methanol, acetaldehyde, acetone, and acetic acid, together with unreacted glycerol, was analyzed by gas chromatography, employing Flame Ionization and Mass Spectrometry detectors (FID and MS) and Total Organic Carbon (TOC). The global conversion of glycerol is calculated as follows:

(1) $$\mathrm{Glycerol}\;\mathrm{conversion}\;(\%)=\frac{N_{\mathrm{glycerol},0}-N_{\mathrm{glycerol}}}{N_{\mathrm{glycerol},0}}\cdot 100$$

where N_glycerol,0_, and N_glycerol_ are the total moles of fed glycerol and total moles of unreacted glycerol, respectively.

The carbon yield to liquids (CYliq) is calculated as the percentage (on a mass basis) of carbon in liquid products (unreacted glycerol excluded), compared to carbon in the glycerol-fed.

(2) $$\mathrm{CYliq}\;(\%)=\frac{\mathrm{mg}C_{\mathrm{liquid}\;\mathrm{products}}}{{\mathrm{mgC}}_{\mathrm{glycerol},0}}\cdot 100$$

The carbon yield to gases (CYgas) is calculated as the percentage of carbon in the gas products (CO_2_, CO, CH_4_, C_2_H_6_, and C_3_H_8_) compared to carbon in the glycerol-fed (on a mass basis).

(3) $$\mathrm{CYgas}\;(\%)=\frac{\mathrm{mg}C_{\mathrm{gas}\;\mathrm{products}}}{{\mathrm{mgC}}_{\mathrm{glycerol},0}}\cdot 100$$

A carbon deficit smaller than 15% has been used, which is acceptable for the reliability of the experiment [11,41]. The carbon deficit is defined as follows:

(4) $$\mathrm{Carbon}\;\mathrm{deficit}=\mathrm{Glycerol}\;\mathrm{conversion}-\left(\mathrm{CYliq}+\mathrm{CYgas}\right)$$

The TON is expressed as the amount of reacted glycerol by each catalyst acid site. The acid sites are defined from two perspectives: (5) specific surface basis and (6) mass basis:

(5) $${\mathrm{TON}}_{\mathrm{surface}}\left(\frac{{\mathrm{mol}}_{\mathrm{gly}}\cdot m^{2}}{{\mathrm{mol}}_{H^{+}}}\right)=\frac{N_{\mathrm{glycerol},0}-N_{\mathrm{glycerol}}}{{\mathrm{TAS}}_{\mathrm{surface}}}$$

(6) $${\mathrm{TON}}_{\mathrm{mass}}\;\left(\frac{{\mathrm{mol}}_{\mathrm{gly}}\cdot g}{{\mathrm{mol}}_{H^{+}}}\right)=\frac{N_{\mathrm{glycerol},0}-N_{\mathrm{glycerol}}}{{\mathrm{TAS}}_{\mathrm{mass}}}$$

where TAS_mass_ refers to the total acid sites expressed as (moles NH_3_/g) and TAS_surface_ refers to the total acid sites expressed as (moles NH_3_/m^2^), being calculated as follows:

(7) $${\mathrm{TAS}}_{\mathrm{surface}}=\frac{{\mathrm{TAS}}_{\mathrm{mass}}}{\mathrm{BET}\;\mathrm{surface}}.$$

Yields of 1,2-PDO, acetol, EtOH and EG, expressed as g/g glycerol, have been calculated as follows:

(8) $$\mathrm{Yield}\;A=\left(\frac{\mathrm{CYliq}}{100}\right)\cdot \left(\frac{S_{A}}{100}\right)\cdot \left(\frac{1}{\mathrm{RA}}\right)\cdot \mathrm{RGlycerol}$$

where RGlycerol is the mass fraction of carbon in glycerol (0.391) and RA is the mass fraction of carbon in compound A.

The carbon selectivity to liquid products, *S_A_*, is defined as the percentage ratio of carbon in a liquid product to the total carbon in all the analyzed liquid products:

(9) $$S_{A}\;(\%)=\frac{\mathrm{mg}C_{\mathrm{liquid}\;\mathrm{product}\;A}}{{\mathrm{mgC}}_{\mathrm{total}\;\mathrm{liquid}\;\mathrm{products}}}\cdot 100$$

## 4. Conclusions

This study shows that the calcination temperature of both the support and the catalyst significantly influences the specific surface area and the CeO_2_ crystallite size. Additionally, for similar amounts of Ni load in the catalysts (i.e., 10 wt.%) the acidity values are directly related to the specific surface area. This effect is also observed in the catalyst performance evaluation in terms of TON_surface_.

The addition of Ni consistently results in the creation of new acid sites, the consequence being the active sites promoting the glycerol dehydration reaction towards acetol, and further acetol hydrogenation to 1,2-PDO. This conclusion is supported by the higher selectivity of this catalyst family for producing liquid products over gaseous products in the APH of glycerol.

The most effective catalysts for glycerol conversion were Ni10/CeO_2_(500)700 (29.0%) and Ni10/CeO_2_(700)700 (25.5%). These catalysts also showed the highest CYliq values of 21.4 and 24.0%, respectively. For all catalysts, the main reaction pathway is acetol/1,2-PDO. Specifically, the Ni10/CeO_2_(700)700 catalyst demonstrated the highest selectivity to this route of approximately 82% (23% corresponding to 1,2-PDO), yielding approximately 0.1136 g acetol/g glycerol and 0.0456 g_1,2-PDO_/g_glycerol_. In contrast, the Ni10/CeO_2_(500)700 catalyst showed values of acetol/1,2-PDO at around 77%, but higher selectivity to 1,2-PDO (33%) and yields to acetol and 1,2-PDO of 0.0758 and 0.058 g/g _glycerol_, respectively. Furthermore, the highest yield to 1,2 PDO (0.0613 g_1,2-PDO_/g_glycerol_) was achieved by the catalyst with the highest Ni content (Ni20/CeO_2_(500)700), which also had the highest TAS_surface_. This confirms the reaction pathway suggested by the authors, in which the acetol reacts with the in situ produced hydrogen towards 1,2-PDO. Furthermore, the acidity effect is also observed in the EG route, enhancing the selectivity towards C–C bond breakage reactions.

Cerium carbonate crystalline phases have been created on the catalysts after the reaction. The catalysts with the highest calcination temperature and consequently higher CeO_2_ sintering (Ni10/CeO_2_(700)700 and Ni10/CeO_2_(500)800) showed less cerium carbonate crystalline phase formation. Nonetheless, these phases showed no relevant influence on the catalytic performance for the 3 h experiment performed in this work.

## Figures and Tables

**Table 1:** Ni content, determined by ICP-OES; textural properties determined by nitrogen adsorption and metallic dispersion, determined by H_2_-chemisorption.

	Ni Content (wt.%)	Surface Area (m^2^/g)	Pore Volume (cm^3^/g)	Pore Diameter (nm)	Metallic Dispersion, D_exp_ (%)
Ni10/CeO_2_(500)600	9.644 ± 0.085	67	0.15	8.8	1.90
Ni10/CeO_2_(500)700	9.793 ± 0.098	54	0.14	10.0	2.72
Ni10/CeO_2_(500)800	9.418 ± 0.066	22	0.07	12.7	1.13
Ni10/CeO_2_(700)700	9.592 ± 0.013	34	0.11	12.9	1.85
Ni20/CeO_2_(500)700	19.670 ± 0.089	38	0.13	13.4	1.62
CeO_2_(500)	-	98	0.23	9.4	-
CeO_2_(700)	-	61	0.19	11.5	-

**Table 2:** Temperature distribution according to the H_2_-TPR profiles deconvolution.

	Temperatures (°C)				
	T1	T2	T3	T4	T5
Ni10/CeO_2_(500)600	172	218	309	335	798
Ni10/CeO_2_(500)700	182	224	320	367	807
Ni10/CeO_2_(500)800	196	204	308	491	810
Ni10/CeO_2_(700)700	160	213	309	413	812
Ni20/CeO_2_(500)700	171	227	341	444	823

**Table 3:** Results of NH_3_-TPD.

	Temperatures (°C)				Strength of Acid Sites * (%)				TAS_mass_	TAS_surface_
	T1	T2	T3	T4	L	L-M	M	H	µmol NH_3_/g	µmol NH_3_/m^2^
CeO_2_(500)	245	413	528	675	-	16	50	34	64	0.65
CeO_2_(700)	241	372	551	740	-	20	57	23	18	0.30
Ni10/CeO_2_(500)600	163	223	346	-	24	67	8	-	196	2.93
Ni10/CeO_2_(500)700	151	238	362	-	32	53	15	-	151	2.80
Ni10/CeO_2_(500)800	145	219	337	-	27	68	5	-	58	2.64
Ni10/CeO_2_(700)700	145	227	367	-	28	56	16	-	78	2.29
Ni20/CeO_2_(500)700	146	231	361	-	34	49	17	-	144	3.79

* L: low; L-M: low-medium; M: medium; H: high.

**Table 4:** Catalytic activity results: global parameters and product distribution to liquids.

	Ni10/CeO_2_(500)600	Ni10/CeO_2_(500)700	Ni10/CeO_2_(500)800	Ni10/CeO_2_(700)700	Ni20/CeO_2_(500)700
Global catalyst performance					
Gly. conv. (%)	19.1 ± 0.9	29.0 ± 0.3	17.1 ± 0.1	25.5 ± 0.1	23.5 ± 0.9
CYliq (%)	15.7 ± 0.8	21.4 ± 0.5	12.6 ± 0.5	24.0 ± 0.1	18.2 ± 0.3
CYgas (%)	1.4 ± 0.2	1.1 ± 0.1	0.8 ± 0.5	1.5 ± 0.9	1.9 ± 0.1
C_def_ (%)	2.0	6.5	3.7	0	3.4
TON_surface_ (mol_gly_·m^2^/mol_H+_)	95	190	98	145	96
TON_mass_ (mol_gly_·g/mol_H+_)	79	175	249	251	139
Selectivity (%)					
1,2-PDO	40 ± 1	33 ± 1	31 ± 1	23 ± 2	41 ± 1
Acetol	39 ± 1	44 ± 2	51 ± 1	59 ± 3	33 ± 1
Acetone	0.54 ± 0.05	0.54 ± 0.01	0.48 ± 0.01	0.82 ± 0.01	0.47 ± 0.03
EG	7.6 ± 0.1	6.3 ± 0.4	6.0 ± 0.1	4.5 ± 0.5	8.4 ± 0.2
EtOH	11.8 ± 0.3	12.4 ± 0.7	9.3 ± 0.1	11.6 ± 0.5	13.8 ± 0.7
Acetic acid	0.9 ± 0.1	3.1 ± 0.1	1.2 ± 0.2	-	2.8 ± 0.3
MeOH	0.9 ± 0.1	0.7 ± 0.1	0.8 ± 0.2	1.2 ± 0.3	0.8 ± 0.1
Yields (g_A_/g_glycerol_)					
1,2-PDO	0.0513	0.058	0.0327	0.0458	0.0613
Acetol	0.0486	0.0758	0.0514	0.1136	0.0484
EG	0.012	0.0136	0.0076	0.0109	0.0154
EtOH	0.0138	0.0198	0.0088	0.0209	0.0188

**Table 5:** Carbon content in the catalyst and metal content in the liquids after the reaction.

	Carbon (wt.%)	Ni (wt.%)	Ce (wt.%)
Ni10/CeO_2_(500)600	3.83	0.39	0.35
Ni10/CeO_2_(500)700	3.88	0.30	0.08
Ni10/CeO_2_(500)800	2.38	0.25	0.07
Ni10/CeO_2_(700)700	1.88	0.35	0.40
Ni20/CeO_2_(500)700	3.96	0.13	0.04

**Table 6:** Catalysts and preparation conditions.

Catalyst	T1 (°C)	T2 (°C)	Nickel Load (wt.%)	Reduction Temp. (°C)
Ni10/CeO_2_(500)600	500	600	10	500
Ni10/CeO_2_(500)700	500	700	10	500
Ni10/CeO_2_(500)800	500	800	10	550
Ni10/CeO_2_(700)700	700	700	10	500
Ni20/CeO_2_(500)700	500	700	20	500

T1: Calcination temperature of the support, T2: Calcination temperature of the catalyst.

## Data Availability

Data are contained within the article and Supplementary Materials.

## Supplementary Materials

The following supporting information can be downloaded from https://www.mdpi.com/article/10.3390/molecules29163797/s1, Adsorption–desorption isotherms of CeO_2_ supports and Ni/CeO_2_ catalysts (Figure S1). Crystallite size of NiO, CeO_2_, and Ni in calcined and reduced catalysts determined by the Scherrer equation (Table S1). Crystallite size results determined for Ni10CeO_2_(500)600, Ni10CeO_2_(500)700, and Ni10CeO_2_(500)800 catalysts (Figure S2). Example of TEM results (Ni10/CeO_2_(500)700—after reduction) (Figure S3). Relationship between the OVD and the catalyst acidity (Figure S4). Comparison of global activity results. TON calculated (1) as a function of acid sites (blue) and (2) as a function of the Ni metallic sites (green) (Figure S5). Selectivity evolution with time for (a) Ni10/CeO_2_(500)700, (b) Ni10/CeO_2_(700)700, and (c) Ni20/CeO_2_(500)700 catalysts (Figure S6).
