# Supplementary material for: Influence of Synthesis Conditions on Catalytic Performance of Ni/CeO2 in Aqueous-Phase Hydrogenolysis of Glycerol without External Hydrogen Input
Source: Molecules. 2024 Aug 10;29(16):3797. doi: 10.3390/molecules29163797 (PMC11357132; doi:10.3390/molecules29163797)
Supplement: Supplementary file 1 [file molecules-29-03797-s001.zip › molecules-3106888-supplementary.pdf]

Influence of synthesis conditions on the catalytic performance of Ni/CeO_2 in Aqueous-Phase Hydrogenolysis of glycerol without external hydrogen input

SUPPLEMENTARY INFORMATION

Catalyst characterization results before the reaction

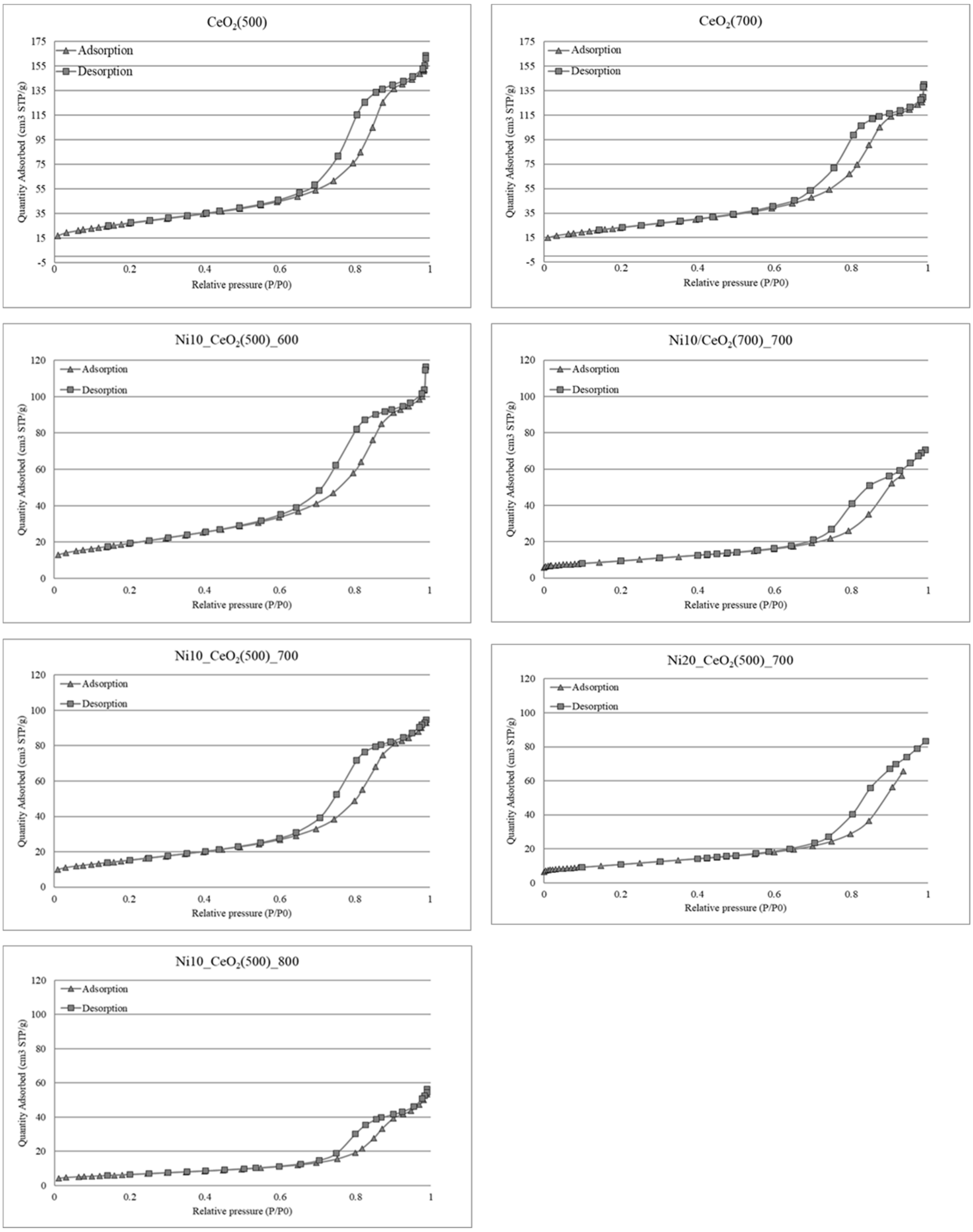

Figure S1. Adsorption-desorption isotherms of CeO_2 supports and Ni/CeO_2 catalysts.

Table S1. Crystallite size of NiO, CeO_2 and Ni in calcined and reduced catalysts, determined by Scherrer equation.

	Calcined catalysts		Reduced catalysts	
	D_{NiO} (nm)	D_{CeO2} (nm)	D_{Ni} (nm)	D_{CeO2} (nm)
Ni10/CeO_2(500)600	26.2	9.8	23.1	9.8
Ni10/CeO_2(500)700	27.8	11.1	21.0	14.7
Ni10/CeO_2(500)800	27.4	18.5	25.5	18.5
Ni10/CeO_2(700)700	30.6	15.4	23.4	16.2
Ni20/CeO_2(500)700	31.9	11.9	29.5	12.3
CeO_2(500)	-	8.8	-	-
CeO_2(700)	-	15.8	-	-

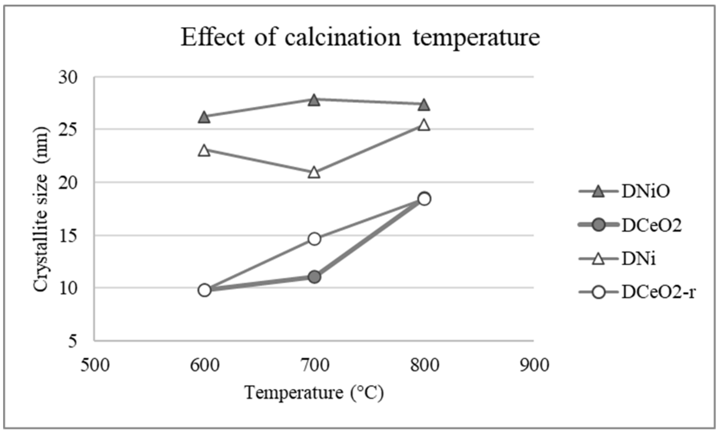

Figure S2. Crystallite size results determined for Ni10CeO_2(500)600, Ni10CeO_2(500)700 and Ni10CeO_2(500)800 catalysts

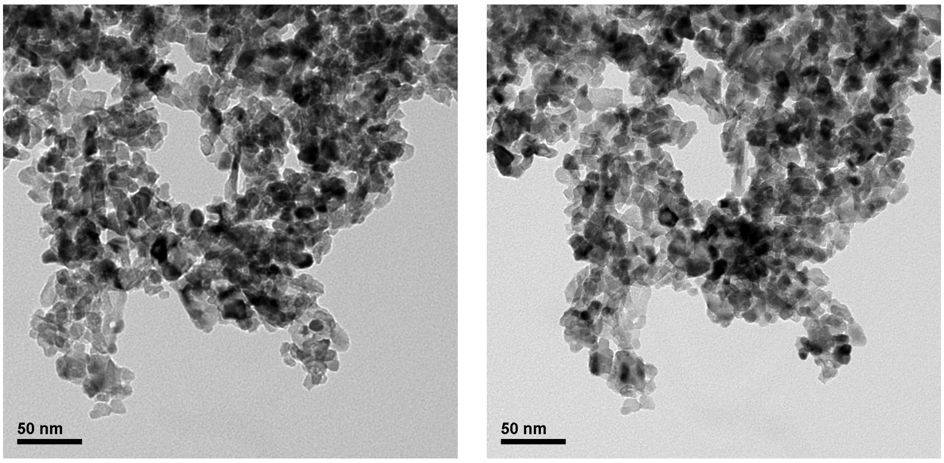

Figure S3. Example of TEM results (Ni10/CeO_2(500)700 – after reduction)

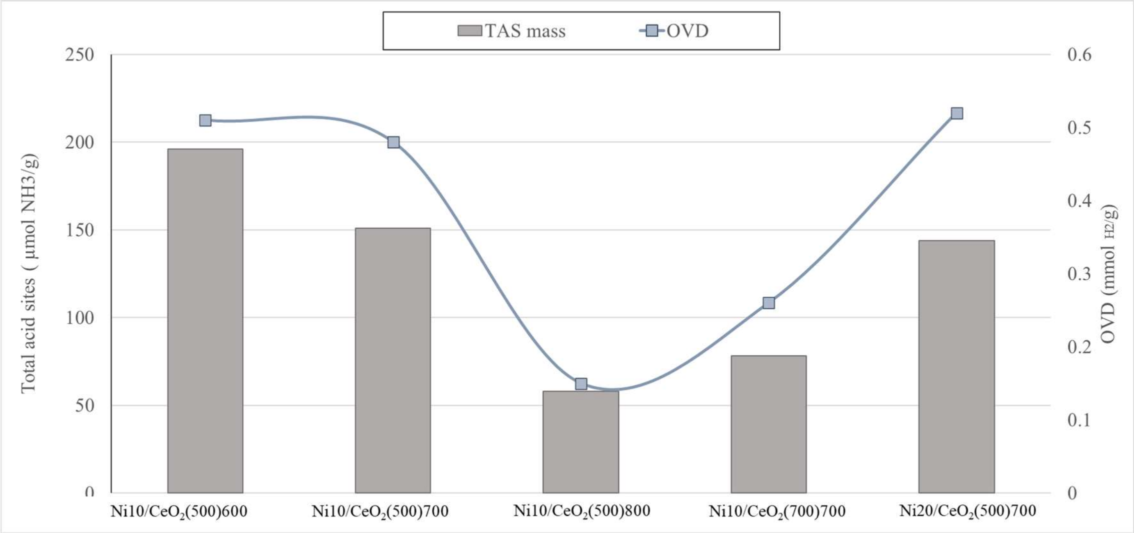

Figure S4. Relationship between the presence of OVD and the total acid sites concentration

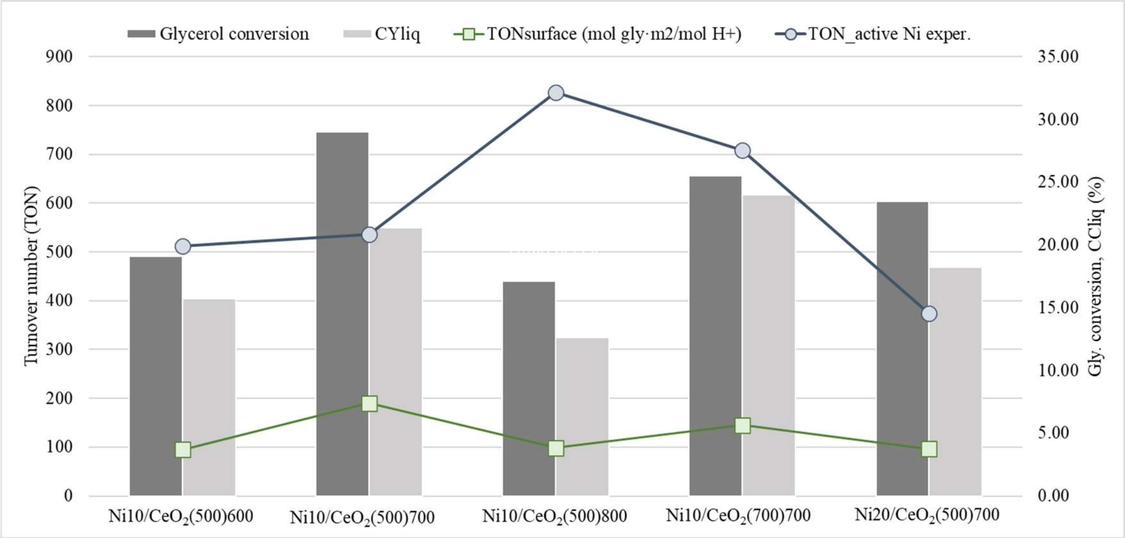

Figure S5. Comparison of global activity results. TON, calculated (1) as a function of acid sites (blue) and (2) as a function of the Ni metallic sites. (green).

$$TON_{active\ Ni\ exper.} = \frac{moles\ reacted\ glycerol}{moles\ Ni\ in\ the\ catalyst \cdot metallic\ dispersion\ (\%)}$$

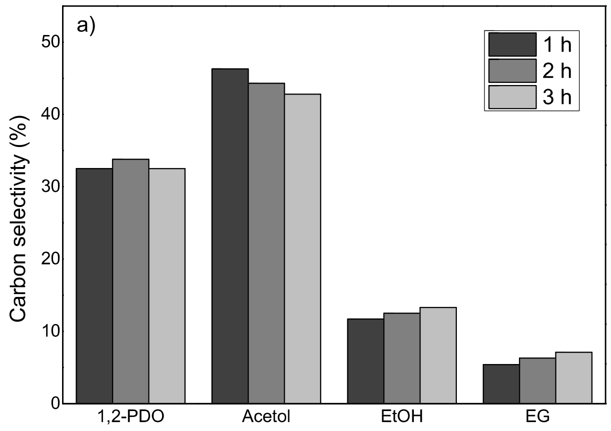

(a)

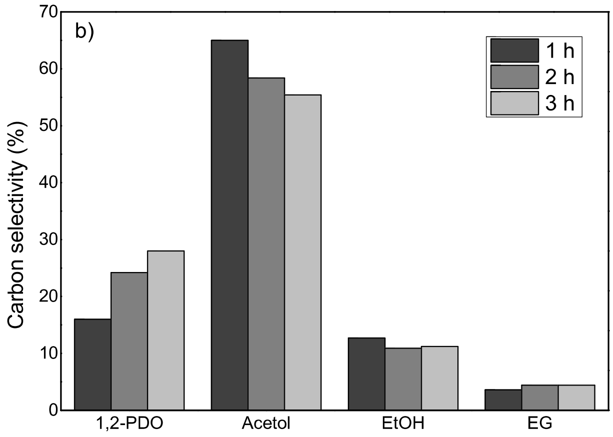

(b)

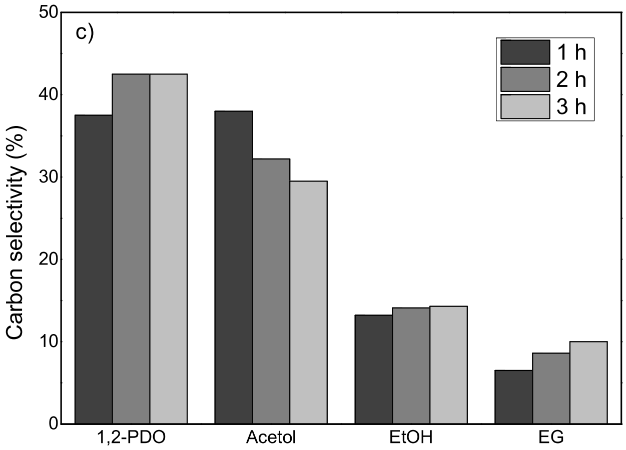

(c)

Figure S6. Selectivity evolution with time for (a) Ni10/CeO_2(500)700, (b) Ni10/CeO_2(700)700 and (c) Ni20/CeO_2(500)700 catalysts, respectively
